# Quantum dot-doped porous silicon metal–semiconductor metal photodetector

**DOI:** 10.1186/1556-276X-7-291

**Published:** 2012-06-06

**Authors:** Chia-Man Chou, Hsing-Tzu Cho, Vincent K S Hsiao, Ken-Tye Yong, Wing-Cheung Law

**Affiliations:** 1 Division of Pediatric Surgery, Department of Surgery, Taichung Veterans General Hospital, 160 sec. 3 Chung-Kang Rd, Taichung, 40705, Taiwan; 2 Department of Applied Materials and Optoelectronic Engineering, National Chi Nan University, No. 1, University Rd, Puli, NanTou, 54561, Taiwan; 3 School of Electrical and Electronic Engineering, Nanyang Technological University, Nanyang, 639798, Singapore; 4 Institute for Lasers, Photonics, and Biophotonics, University at Buffalo, The State University of New York, Buffalo, NY, 14260-4200, USA

**Keywords:** Porous silicon, Quantum dots, Photodetector

## Abstract

In this paper, we report on the enhancement of spectral photoresponsivity of porous silicon metal–semiconductor metal (PS-MSM) photodetector embedded with colloidal quantum dots (QDs) inside the pore layer. The detection efficiency of QDs/PS hybrid-MSM photodetector was enhanced by five times larger than that of the undoped PS-MSM photodetector. The bandgap alignment between PS (approximately 1.77 eV) and QDs (approximately 1.91 eV) facilitates the photoinduced electron transfer from QDs to PS whereby enhancing the photoresponsivity. We also showed that the photoresponsitivity of QD/PS hybrid-MSM photodetector depends on the number of layer coatings of QDs and the pore sizes of PS.

## **Background**

Colloidal quantum dots (QDs) are semiconductor nanocrystals with tunable optical property depending on their sizes and shapes that can be controlled by the fabrication process. Outstanding optoelectronic and photonic properties, such as electroluminescence, photoluminescence, photovoltaics, absorption, and narrow emission linewidth, make QDs a good candidate for photodetectors [1-6], light emitting diodes [7-9] and lasers applications [10-12]. Because of the QDs’ inherent solution-processable property, several studies have incorporated QDs into organic or inorganic matrix and demonstrated these hybrid devices with improved optoelectronic performances [13-20]. In this paper, we report on the enhancement of photoresponsivity in inorganic/inorganic hybrid device consisting of porous silicon (PS)-MSM (metal–semiconductor–metal) photodetector and CdSe/CdS/ZnS core shell QDs embedded in the pores within the PS. The bandgap alignment between PS (approximately 1.77 eV) and QDs (approximately 1.91 eV) facilitates the photoinduced electron transfer from QDs to PS and further enhances the photoresponsivity. We also show that the photoresponsitivity depends on the numbers of coating of QDs and the pore size of PS which could be controlled by the anodization time.

## **Methods**

**Figure 1:**
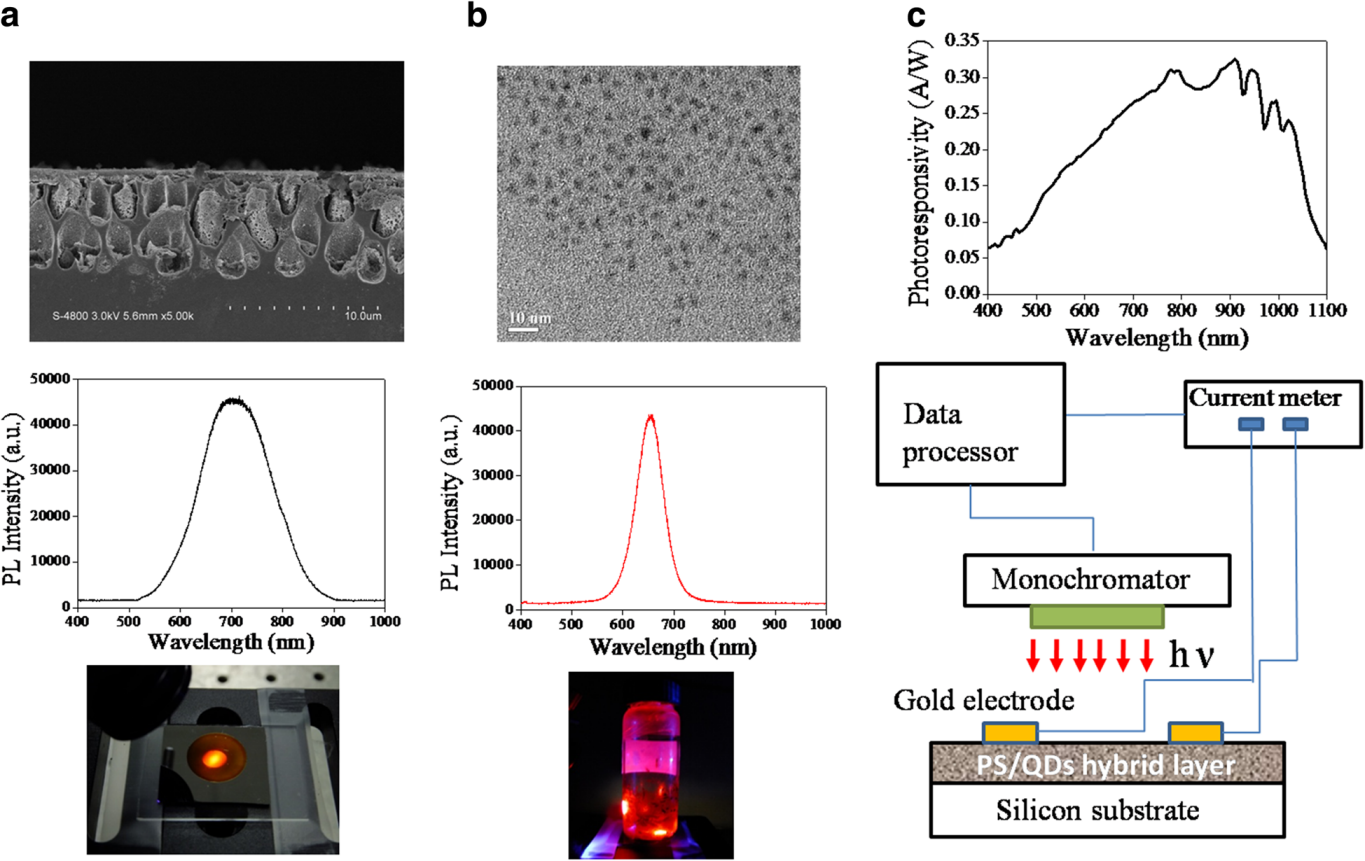
**Electron microscopic images, and PL spectrum and images of PS and QDs.** (**a**) Cross-sectional SEM image of PS and corresponded PL spectrum and emission image. (**b**) Transmission electron microscopy (TEM) image of ZnS/CdSe core shell QDs and corresponded PL spectrum and emission image. (**c**) Schematic setup of spectral photoresponsivity measurement.

The PS layers were fabricated by anodic etching where a p-type silicon substrate was placed in the homemade Telflon (E.I. du Pont de Nemours and Company, Wilmington, DE, USA) etching cell using a mixture of aqueous hydrogen fluoride (purity 49%) and ethanol (purity 99%), 1:1 by volume. The sample was anodized at current density of 16 mA/cm^2^ and at different time. No further chemical or thermal treatment was carried after etching. A stainless steel shadow mask with the MSM finger pattern of spacing and width of about 100 and 100 μm, respectively, was fabricated by micromachining process. The MSM finger pattern was formed by Au metal thermal evaporation. The CdSe/CdS/ZnS core-shell colloidal QDs absorbed on the internal pores within PS were then prepared by dipping the PS using CdSe/CdS/ZnS QDs (0.6 mg) dissolved in toluene (100 mg) and evaporating toluene at room temperature for 30 min. Figure 1a shows the cross-sectional image of anodized PS captured by scanning electronic microscopy (SEM). The average pore depth is about 8 μm. Figure 1a, middle image, also show the photoluminescence (PL) spectrum and fluorescence emission images of PS, indicating a PL emission wavelength of approximately 700 nm (approximately 1.77 eV). The CdSe/CdS/ZnS core-shell colloidal QDs were prepared by growing a CdS/ZnS shell on a CdSe core [21]. For core CdSe QDs, they can be synthesized at 300°C by the reaction between cadmium oxide dissolved in oleic acid and trioctylphosphine (TOP) and TOPSe. The reaction resulted in the formation of monodispersed CdSe QDs. The shell growth of CdS and ZnS was uniform and epitaxial and eventually coats onto the CdSe core. The average diameter of core shell QDs was approximately 5 nm, as shown in Figure 1b. Figure 1b also shows the PL spectrum and image of fluorescence emission of QDs, indicating a PL emission wavelength of approximately 650 nm (approximately 1.99 eV). Photoresponsivity measurements were performed at room temperature employing a light source (50 W halogen lamp) equipped with a monochromator (Acton SP2155, Princeton Instruments Inc., Trenton, NJ, USA) and a current meter (Keithley 6485, Keithley Instruments Inc., Cleveland, OH, USA), as shown in Figure 1c. Figure 1c also shows the spectral photoresponstivity of standard silicon-based photodetector (SI 1337). The spectral photoresponsivity of fabricated MSM photodetector was determined relatively to the value measured from the standard silicon-based photodetector. 

## **Results and discussion**

**Figure 2:**
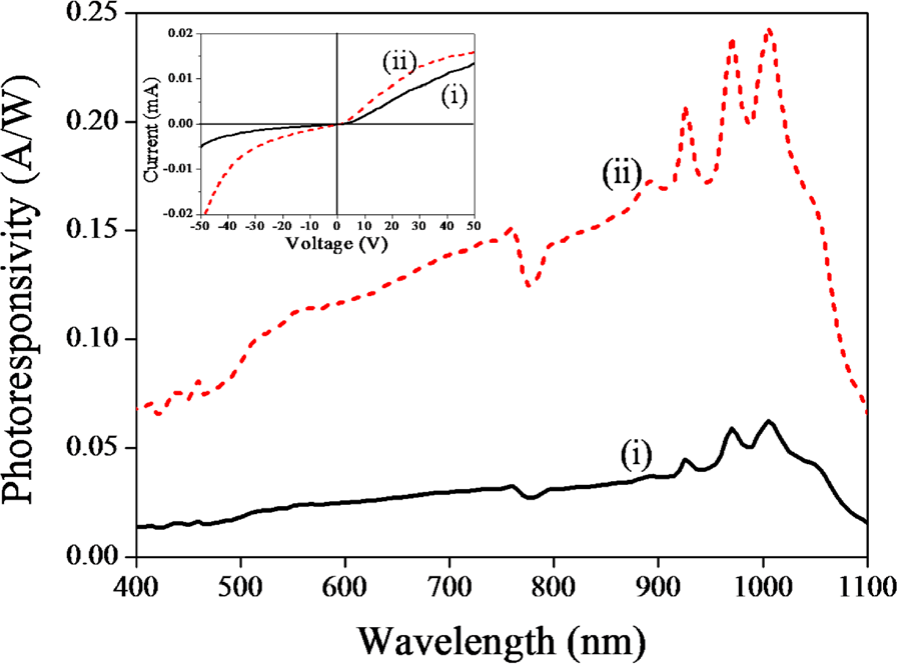
**Comparison of spectral photoresponsivity and*I-V*curve from QD-doped and undoped samples.** Comparison of spectral photoresponsivity and *I-V* curve (inset) from undoped (curve i) and QDs doped (curve ii) PS-MSM photodetector. The anodization time was 25 min. *I-V* curve was measured at dark environment.

**Figure 3:**
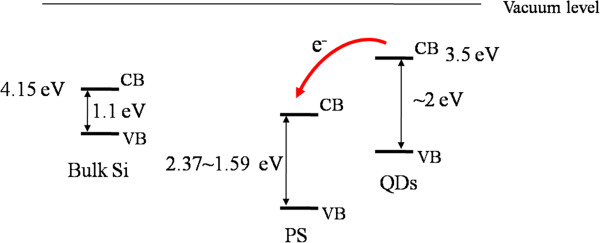
Proposed model for electron transfer at the PS/QD interface.

Figure 2 (inset) shows the dark current of a PS-MSM photodetector before, curve (i), and after, curve (ii), doping QDs characterized by the *I-V* measurement. The increase of dark current indicates that the doped QDs enhance the charge transfer between PS and electrode. We speculate that the QDs may provide a shunt conduction path next to the PS that might explain the increase of the dark current. As expected from the symmetry of the MSM structure, the *I-V* characteristics in our current device should be symmetric; however, the *I-V* curve measured here are not perfect symmetry around zero. The discrepancy may be due to the inhomogeneous etching within the area (10 × 10 mm^2^) of MSM device. Figure 2 shows that the photoresponsivity increases in doped QDs/PS hybrid-MSM photodetector comparing to undoped PS-MSM photodetector. Figure 3 proposes a model for electron transfer at the PS/QD interface in a QDs/PS hybrid-MSM photodetector sample. The bulk Si has indirect bandgap, but porous PS has direct bandgap. The energy-band structure of PS could be predicted by PL measurement of PS samples [22]. The direct bandgap semiconductors offer much stronger absorption coefficient; therefore, the PS has better optoelectronic properties than bulk Si. After adding QDs onto the PS sample, the band alignment between the two materials allows photoinduced electron transfer from the QDs to the PS. In addition, QD nanostructures could provide large surface areas and short diffusion lengths for photogenerated charge carriers [23]. The photoresponsivity behavior of QDs/PS hybrid-MSM photodetector was recorded by comparing to the photoresponsivity of the standard silicon-based photodetector, as shown in the inset from Figure 1c, so the enhancement of photoresponsivity from QDs/PS hybrid-MSM photodetector has maximum photoresponsivity at approximately 900 nm. 

**Figure 4:**
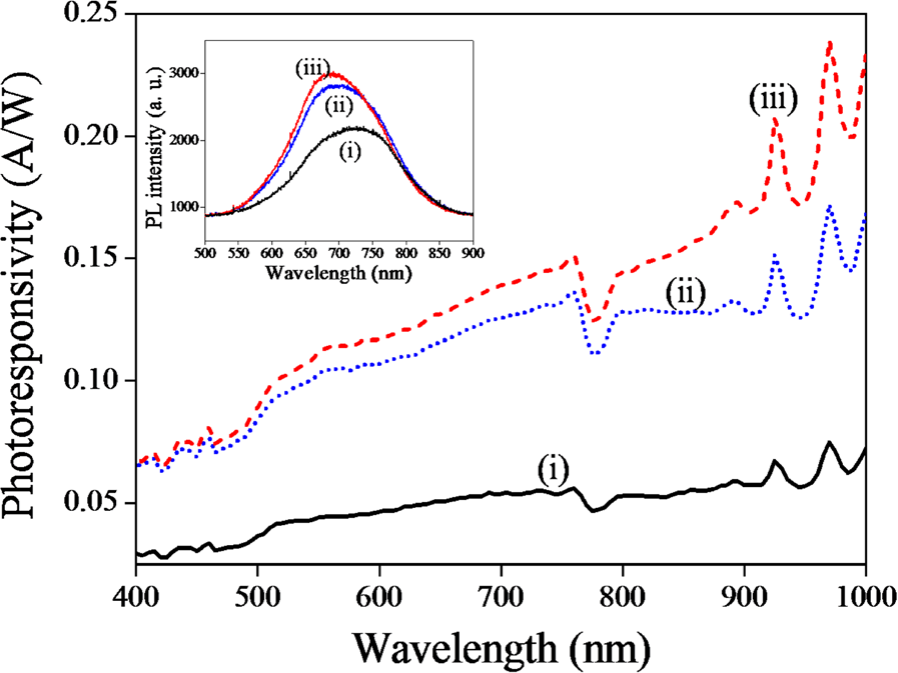
**Comparison of spectral photoresponsivity and PL spectrum at different PS anodization times.** Spectral photoresponsivity and PL spectrum (inset) of QDs/PS hybrid-MSM photodetector dependent on anodization times, 5 (i), 10 (ii) and 25 (iii) min, of porous silicon at 16 mA/cm^2^ anodization current.

The anodization time of PS is an important parameter for the PS-MSM photodetector. Figure 4 shows spectral photoresponsivity of QDs/PS hybrid-MSM photodetector for various anodization times. The anodization current density was 20 mA/cm^2^. It is obvious that the photoresponsivity increases with increasing the anodization time. Figure 4 (inset) shows the PL spectrum dependent on anodization time. We observed that the peak wavelength within PL spectrum shifts from 720 to 700 nm indicating the energy bandgap of PS changes from 1.72 to 1.77 eV. Higher PS bandgap energy (1.77 eV) favors the bandgap alignment between PS and QDs (1.91 eV) and further enhances the photoresponsivity of QDs/PS hybrid-MSM photodetector.

**Figure 5:**
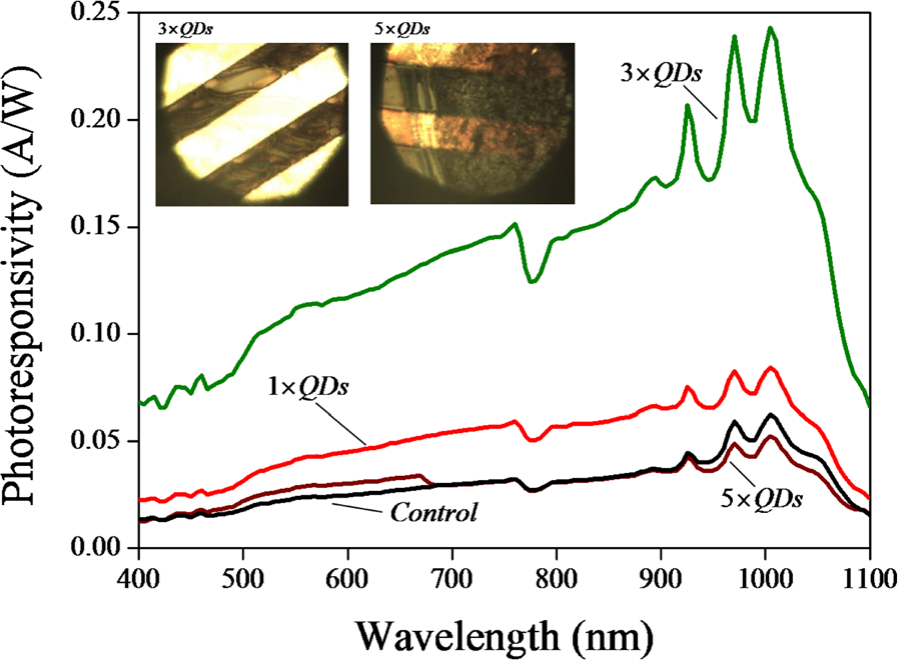
**Spectral photoresponsivity dependent on the number of QD coatings.** Spectral photoresponsivity and optical microscopic image (inset) of QDs/PS hybrid MSM photodetector dependent on the number of coatings. The anodization time was 25 min.

Figure 5 shows the spectral photoresponsivity for the PS-MSM photodetector (control) and QDs/PS hybrid-MSM photodetector obtained after one, three, and five coatings. The photoresponsivity increases with increasing QD coating layers and reaches at maximum value when three coating layer processes was applied. However, we observed that the photoresponsivity decreases when five coatings process are applied on the PS-MSM photodetector. Figure 5 (inset) compares the optical microscopic images of the MSM device after different numbers of QD coating process. The yellow bright region is the electrode while the dark brown region is the PS. We speculate that five-time coating processes make the QDs aggregate together and decrease the photoresponsivity.

## **Conclusions**

We have presented an enhancement of the photoresponsitivity of QDs/PS hybrid MSM photodetector. We found out that the bandgap alignment between QDs and PS facilitates the electron transfer and further increases the photoresponsitivity. By choosing different anodization time and layers of QD coating, we demonstrate the flexibility in fabricating QDs/PS hybrid MSM photodetector.

## **Competing interests**

The authors declare that they have no competing interests.

## **Authors’ contributions**

VKSH proposed the idea and helped in the preparation of the manuscript. CMC and HTC designed and carried out the experiment, analyzed the data, and prepared the manuscript. KTY and WCL carried out the fabrication of QDs and provided a helpful discussion. All authors read and approved the final manuscript.

## **Authors’ information**

VKSH received a Ph.D. from Electrical Engineering in SUNY at Buffalo in 2005. He is currently an associate professor in the Department of Applied Materials and Optoelectronic Engineering at National Chi Nan University, Taiwan. His research interests include organic/inorganic nanoporous materials, photoresponsive LC-based photonic devices, and optical thin films. CMC is a medical doctor at the Department of Surgery in Taichung Veterans General Hospital, Taiwan. HTC is an undergraduate student. KTY is an assistant professor at the Nanyang Technological University in the School of Electrical and Electronic Engineering. He received his Ph.D. from Chemical and Biological Engineering in SUNY at Buffalo in 2006. His research interests involve the synthesis, functionalization, and application of nanoparticles. WCL is a postdoctoral scholar in the Institute for Lasers, Photonics and Biophotonics (ILPB).

## References

[B1] McDonald SA Konstantatos G Zhang S Cyr PW Klem EJD Levina L Sargent EH Solution-processed PbS quantum dot infrared photodetectors and photovoltaics Nat Mater 2005 4 138 142 10.1038/nmat1299 15640806

[B2] Konstantatos G Howard I Fischer A Hoogland S Clifford J Klem E Levina L Sargent EH Ultrasensitive solution-cast quantum dot photodetectors Nature 2006 442 180 183 10.1038/nature04855 16838017

[B3] Shieh J-M Yu W-C Huang JY Wang C-K Dai B-T Jhan H-Y Hsu C-W Kuo H-C Yang F-L Pan C-L Near-infrared silicon quantum dots metal-oxide-semiconductor field-effect transistor photodetector Appl Phys Lett 2009 94 241108 10.1063/1.3156806

[B4] Tu C-C Tang L Huang J Voutsas A Lin LY Solution-processed photodetectors from colloidal silicon nano/micro particle composite Opt Express 2010 18 21622 21627 10.1364/OE.18.021622 20941060

[B5] Nayfeh OM Rao S Smith A Therrien J Nayfeh MH Thin film silicon nanoparticle UV photodetector IEEE Photo 2004 16 1927 1929 10.1109/LPT.2004.831271

[B6] Passmore BS Jiang Wu Kunets VP Lytvyn PM Salamo GJ Manasreh MO Room temperature near-infrared photoresponse based on interband transitions in In0.35Ga0.65As multiple quantum dot photodetectors Electron Device Letters 2008 29 224 227

[B7] Dabbousi BO Bawendi MG Onitsuka O Rubner MF Electroluminescence from CdSe quantum-dot/polymer composites Appl Phys Lett 1995 66 1316 1318 10.1063/1.113227

[B8] Hikmet RAM Chin PTK Talapin DV Weller H Polarized-light-emitting quantum-rod diodes Adv Mater 2005 17 1436 1439 10.1002/adma.200401763 34412435

[B9] Jiang Wu Wang ZM Dorogan VG Li S Li Z Mazur YI Salamo GJ Near infrared broadband emission of In0.35Ga0.65As quantum dots on high index GaAs surfaces Nanoscale 2011 3 1485 1488 10.1039/c0nr00973c 21384043

[B10] Reitzenstein S Bazhenov A Gorbunov A Hofmann C Munch S Loffler A Kamp M Reithmaier JP Kulakovskii VD Forchel A Lasing in high-Q quantum-dot micropillar cavities Appl Phys Lett 2006 89 051107 10.1063/1.2266231

[B11] Gao S Zhang C Liu Y Su H Wei L Huang T Dellas N Shang S Mohney SE Wang J Lasing from colloidal InP/ZnS quantum dots Opt Express 2011 19 5528 5535 10.1364/OE.19.005528 21445191

[B12] Shchekin OB Deppe DG 1.3 μm InAs quantum dot laser with To = 161K from 0 to 80°C Appl Phys Lett 2002 80 3277 10.1063/1.1476708

[B13] Hoyer P Koenkamp R Photoconduction in porous TiO_2_ sensitized by PbS quantum dots Appl Phys Lett 1995 66 349 351 10.1063/1.114209

[B14] Huynh WU Dittmer JJ Alivisatos AP Hybrid nanorod-polymer solar cells Science 2002 295 2425 2427 10.1126/science.1069156 11923531

[B15] Alguno A Usami N Ujihara T Fujiwara K Sazaki G Nakajima K Enhanced quantum efficiency of solar cells with self-assembled Ge dots stocked in multilayer structure Appl Phys Lett 2003 83 1258 1260 10.1063/1.1600838

[B16] Choudhury KR Sahoo Y Prasad PN Hybrid quantum-dot-polymer nanocomposites for infrared photorefractivity at an optical communication wavelength Adv Mater 2005 17 2877 2881 10.1002/adma.200501489

[B17] Li X Chon JWM Evans RA Gu M Quantum-rod dispersed photopolymers for multi-dimensional photonic applications Opt Express 2009 17 2954 2961 10.1364/OE.17.002954 19219199

[B18] Shieh J-M Huang JY Yu W-C Huang J-D Wang Y-C Chen C-W Wang C-K Huang W-H Cho A-T Kuo H-C Dai B-T Yang F-L Pan C-L Nonvolatile memory with switching interfacial polar structures of nano Si-in-mesoporous silica Appl Phys Lett 2009 95 143501 10.1063/1.3240888

[B19] Huang JY Shieh JM Kuo HC Pan CL Interfacial polar-bonding-induced multifunctionality of nano-silicon in mesoporous silica Adv Funct Mater 2009 19 2089 2094 10.1002/adfm.200801336

[B20] Qiao H Guan B Böcking T Gal M Gooding JJ Reece PJ Optical properties of II-VI colloidal quantum dot doped porous silicon microcavities Appl Phys Lett 2010 96 161106 10.1063/1.3404183

[B21] Qian J Yong K-T Roy I Ohulchanskyy TY Bergey EJ Lee HH Tramposch KM He S Maitra A Prasad PN Imaging pancreatic cancer using surface-functionalized quantum dots J Phys Chem B 2007 111 6969 6972 10.1021/jp070620n 17552555

[B22] Egeberg RC Veje E Ferreira Da Silva A Pepe I Santos Alves A The energy-band structure of porous silicon studied with photoluminescence excitation and photoacoustic spectroscopy J. Porous Mater 2000 7 173 176 10.1023/A:1009674401781

[B23] Tang J Sargent EH Infrared Colloidal Quantum Dots for Photovoltaics: Fundamentals and Recent Progress Adv Mater 2011 23 12 29 10.1002/adma.201001491 20842658

